# The role of the Xist 5’ m6A region and RBM15 in X chromosome inactivation

**DOI:** 10.12688/wellcomeopenres.15711.1

**Published:** 2020-02-17

**Authors:** Heather Coker, Guifeng Wei, Benoit Moindrot, Shabaz Mohammed, Tatyana Nesterova, Neil Brockdorff

**Affiliations:** 1 Developmental Epigenetics, Department of Biochemistry, University of Oxford, South Parks Road, Oxford, OX1 3QU, UK; 2 Université Paris-Saclay, CEA, CNRS, Institute for Integrative Biology of the Cell (I2BC), Gif-sur-Yvette, 91198, France; 3 Proteomics Technology Development and Application, Department of Biochemistry, University of Oxford, South Parks Road, Oxford, OX1 3QU, UK

**Keywords:** X chromosome inactivation, Xist RNA, m6A, Rbm15

## Abstract

**Background**: X chromosome inactivation in mammals is regulated by the non-coding (nc) RNA, Xist, which represses the chromosome from which it is transcribed.  High levels of the N6-methyladenosine (m6A) RNA modification occur within Xist exon I, close to the 5’ end of the transcript, and also further 3’, in Xist exon VII. The m6A modification is catalysed by the METTL3/14 complex that is directed to specific targets, including Xist, by the RNA binding protein RBM15/15B. m6A modification of Xist RNA has been reported to be important for Xist–mediated gene silencing.

**Methods**: We use CRISPR/Cas9 mediated mutagenesis to delete sequences around the 5’ m6A region in interspecific XX mouse embryonic stem cells (mESCs).  Following induction of Xist RNA expression, we assay chromosome silencing using allelic RNA-seq and Xist m6A distribution using m6A-seq. Additionally, we use Xist RNA FISH to analyse the effect of deleting the 5’ m6A region on the function of the endogenous Xist promoter. We purify epitope tagged RBM15 from mESCs, and then apply MS/MS analysis to define the RBM15 interactome.

**Results**: We show that a deletion encompassing the entire Xist 5’ m6A region results in a modest reduction in Xist-mediated silencing, and that the 5’ m6A region overlaps essential DNA elements required for activation of the endogenous Xist promoter. Deletion of the Xist A-repeat, to which RBM15 binds, entirely abolishes deposition of m6A in the Xist 5’ m6A region without affecting the modification in exon VII. We show that in mESCs, RBM15 interacts with the m6A complex, the SETD1B histone modifying complex, and several proteins linked to RNA metabolism.

**Conclusions**: Our findings support that RBM15 binding to the Xist A-repeat recruits the m6A complex to the 5’ Xist m6A region and that this region plays a role in Xist-mediated chromosome silencing.

## Introduction

X chromosome inactivation (XCI) is the mechanism that evolved in mammals to equalise levels of X-linked gene expression in XX females relative to XY males (reviewed in Heard *et al.*, 1997). The XCI process is regulated by a 17kb non-coding RNA, Xist (reviewed in Gendrel & Heard, 2014). Xist RNA is expressed from the future inactive X chromosome (Xi) in cells of early embryos, and accumulates in cis over the length of the chromosome from which it is transcribed, triggering recruitment of factors that modify the underlying chromatin and repress gene transcription.

Recent studies identified key RNA binding proteins (RBPs) that function in Xist-mediated chromosome silencing ( Chu *et al.*, 2015; McHugh *et al.*, 2015; Minajigi *et al.*, 2015; Moindrot *et al.*, 2015; Monfort *et al.*, 2015). Amongst these are the related RBPs SPEN and RBM15, both of which have been reported to bind to a tandemly repeated element at the 5’ end of Xist, the A-repeat ( Cirillo *et al.*, 2016; Lu *et al.*, 2016; Monfort *et al.*, 2015; Patil *et al.*, 2016), shown in previous work to be the critical element required for Xist-mediated chromosome silencing ( Wutz *et al.*, 2002). SPEN directs recruitment of the NCoR-HDAC3 corepressor complex, which catalyses histone deacetylation, and this is thought to account for its function in XCI, at least in part ( McHugh *et al.*, 2015). RBM15, on the other hand, has been shown to interact with the METTL3/14 complex that catalyses the N6-methyladenosine (m6A) modification on mRNA ( Horiuchi *et al.*, 2013; Patil *et al.*, 2016). Consistent with this observation, transcriptome-wide m6A mapping analysis has revealed a major site of m6A deposition immediately 3’ of the A-repeat in mouse ( Linder *et al.*, 2015; Nesterova *et al.*, 2019) and human Xist/XIST RNA ( Dominissini *et al.*, 2012; Patil *et al.*, 2016). Other heavily m6A-modified sites on Xist RNA include a region in exon VII of mouse Xist RNA, 3’ of another tandem repeat element, the E-repeat.

Building on initial evidence that RBM15 and the protein WTAP, a regulatory subunit of the METTL3/14 complex, play a role in Xist-mediated chromosome silencing, Patil *et al.* (2016) reported that depletion of RBM15 and the homologous protein RBM15B, or of METTL3, the core catalytic subunit of the m6A complex, strongly abrogates Xist-mediated silencing. It was further reported that YTHDC1, a nuclear protein that recognises and binds to m6A sites on RNA, is critically required for chromosome silencing by Xist RNA, a finding that was substantiated by tethering the protein to Xist transcripts in the absence of m6A methylation. Set against these findings, we recently performed a systematic analysis of different factors implicated in Xist-mediated chromosome silencing in mouse embryonic stem cells (mESCs), and found that while RBM15 and the m6A complex do play a role, the magnitude of the effect is relatively modest ( Nesterova *et al.*, 2019).

The interpretation of experiments that perturb the function of the METTL3/14 complex are complicated by the fact that the m6A modification has a fundamental role in RNA metabolism and translation ( Yue *et al.*, 2015; Zaccara *et al.*, 2019), and as such, effects on Xist-mediated silencing could be indirect and/or independent of m6A modification of Xist transcripts. To address this issue, we previously reported that overlapping partial deletions within the major 5’ m6A region in Xist RNA have little or no effect on Xist-mediated silencing ( Nesterova *et al.*, 2019). To extend these findings, we show here that a larger deletion encompassing the entire 5’ m6A region similarly has only a small effect on Xist-mediated silencing. In related experiments we show the 5’ m6A region overlaps with the major Xist enhancer required for Xist gene activation during normal development, providing an explanation for previous reports implicating this region in Xist promoter activity ( Hoki *et al.*, 2009; Royce-Tolland *et al.*, 2010). Additionally, we show that the Xist A-repeat is critical for m6A deposition at the exon I m6A region, but not at the exon VII peak. Finally, we analyse the RBM15 interactome in mESCs, demonstrating strong association with the m6A complex and several other factors, including, as observed previously, the SET1B complex that catalyses histone H3K4 methylation ( Lee & Skalnik, 2012).

## Methods

### ES cell culture

ES cells were grown in Dulbecco’s Modified Eagle Medium (DMEM, Life Technologies) supplemented with 10% foetal calf serum (Seralab), 2 mM L-glutamine, 0.1 mM non-essential amino acids, 50 µM β-mercaptoethanol, 100 U/ml penicillin / 100 µg/ml streptomycin (all Life Technologies) and 1000 U/ml leukemia inhibitory factor (LIF, made in-house). All ES cells were grown on gelatinised plates at 37°C in a humid atmosphere with 5% CO _2_. XT67E1 ES cells were grown in feeder-free conditions, whilst iXist-ChrX ESC and emGFP-PreScission-RBM15 ESC were grown using Mitomycin C-inactivated mouse fibroblasts as feeders. Xist expression was induced by the addition of 1–1.5 µg/mL doxycycline (Dox) (Sigma, D9891) for 24 hrs.

Differentiation of XT67E1 ES cells was achieved using retinoic acid (RA) for three days. Briefly, cells were seeded at 2.5 × 10 ^4^ cells/cm ^2^ and allowed to attach overnight in ES media. ES media without LIF but containing a final concentration of 10 ^-6^ M RA (Sigma) was used to culture the cells subsequently for a further two days, when the cells were seeded onto coverslips for RNA-FISH. After a third day cultured with RA, cells were then cultured for a fourth day in ES media without LIF or RA and then harvested.

### Generation of mES cell lines

The XT67E1 m6A deletion XX ES line (129/PGK) was derived from cells described previously ( Penny *et al.*, 1996), and in which Xist is only expressed from the PGK allele. Here, the functional PGK-derived Xist allele was targeted using CRISPR-mediated homologous recombination: cells were transfected using Lipofectamine 2000 (Life Technologies) according to the manufacturer’s instructions with equimolar amounts of the sgRNA-expressing plasmid (0.85 ug, pX459v2-HC_Xist1_843; 5’ CTTAAACTGAGTGGGTGTTC 3’) together with the targeting vector (1.5 ug pBSK_XistEV_deltam6A), containing homology arms 892 bp upstream and 1419 bp downstream of the 177 bp deletion of Xist. After 18 hrs transfected cells were passaged to 90 mm gelatinised Petri dishes and 1.5 μg/mL puromycin applied 5 hrs later. Cells were grown under puromycin selection for two days and then without puromycin for a further 6–8 days, until colonies were picked. Selected clones were screened for deletion of the m6A-containing region using PCR (Eppendorf flexlid Mastercycler nexus GX2 Gradient Eco PCR machine) with primers flanking the deleted genomic DNA (forward primer, Dbl m6A targeting Primer1F: 5’ TTTTTTTTCACGGCCCAACGGGGCG 3’ and reverse primer, Dbl m6A targeting_Primer2R: ATACCGCACCAAGAACTTGAGCC), Invitrogen Taq DNA Polymerase (18038-042) and cycling conditions of 94°C for 2min, followed by 30 cycles comprised of 94°C for 30 sec, 55°C for 30 sec, 72°C for 1 min, then finally 72°C for 5 min before being validated by Sanger sequencing.

To generate the iXist-ChrX_11C m6A deletion ES line, interspecific (129/Sv-Cast/Ei) XX ES cells with a tetracycline-inducible promoter on the *M.m.domesticus* Xist allele and rtTA expressed from the TIGRE locus (described in detail in Nesterova *et al.* (2019)), were further modified using CRISPR-mediated homologous recombination as detailed above, using the sgRNA expressing plasmid (2 ug, pX459v2-HC_Xist1_843; CRISPR target: 5’ CTTAAACTGAGTGGGTGTTC 3’) and targeting vector (2 ug, pBSK_XistEV_fulldeltam6A, containing homology arms 815 bp upstream and 1251 bp downstream of the 355 bp deletion of Xist). After 18 hrs transfected cells were passaged to 90 mm gelatinised Petri dishes with feeders. Puromycin selection and PCR screening was carried out as detailed above, and clones validated by Sanger sequencing.

To generate the iXist-ChrX_A_2 ES cell lines containing a precise deletion of the Xist A-repeat region, CRISPR-mediated homologous recombination was performed in iXist-ChrX cells as described above. Briefly, cells were transfected with 1 μg of each sgRNA (Plasmid 1703_sg_Xist_TNK404_2A-PuroV2; 5’ ttttttttCACGGCCCAACG 3’ and Plasmid 1704_sg_Xist_TNK410_2A-PuroV2; 5’ tccttagcccatcggggcca 3’) and 3 μg of targeting vector (Plasmid 1705_pBS_Xist_delA_dom, containing 328bp (5’) and 385bp (3’) homology regions surrounding the A-repeats. Puromycin selection was applied 48 hrs after transfection for two days. Clones were identified by PCR screening and Sanger sequencing and further validated by Southern blot.

The emGFP-PreScission-RBM15 cell line was derived from XY 3E ES cells, containing rtTA integrated into the Rosa26 locus and random integration of Dox-inducible Xist transgene into chr17 ( Tang *et al.*, 2010). In these cells, the puromycin resistance cassette at Rosa26 locus was replaced with hygromycin resistance ( Moindrot *et al.*, 2015). Then, cells were transfected and screened for stable integration of the pTRE-emGFP-PreScission-RBM15 plasmid. Cells treated with 1µg/mL Dox for 24hr simultaneously induce Xist RNA and emGFP-PreScission-RBM15 protein expression.

### RNA-FISH

Cells were plated on 22mm ^2^ glass coverslips and grown overnight. After 24 hr Xist induction, coverslips were washed twice with PBS, fixed using 3.7 % formaldehyde for 10 min at room temperature and then after a brief PBS wash, permeabilised for 10 min with 0.5% Triton X-100 (Sigma) at room temperature. Coverslips were then washed two times in 70% ethanol and either stored in 70% ethanol at 4°C until use or dehydrated (80%, 95%, 100% ethanol, 5 min each, RT) and air dried, before overnight hybridisation at 37°C in a humid chamber with Xist probe, diluted in 2X hybridisation buffer (5X SSC, 12.5% dextran sulfate, 2.5 mg / mL BSA (NEB)). The Xist probe was generated from an 18 kb cloned cDNA spanning the whole Xist transcript as previously described ( Moindrot *et al.*, 2015; Nesterova *et al.*, 2019). After incubation, slides were washed three times for 5 minutes each with 2X SSC / 50% formamide at 42°C followed by three washes of 5 minutes each with 2X SSC at 42°C. Slides were mounted with Vectashield containing DAPI (Vector labs) and sealed with nail varnish. Coverslips were visualised using a 63X oil immersion objective and a Zeiss Axio Observer Z1 microscope.

### Co-immunoprecipitation (co-IP) assays

Nuclear extracts were prepared using mESC grown without feeders for the last passage, as described in ( Pintacuda *et al.*, 2017) using Benzonase. The coupling of in-house GFP-nanobodies to M-280 Tosyl-activated Dynabeads (Thermo-Fisher; #14204) was performed as described in ( Pintacuda *et al.*, 2017). Co-IP assays were performed in mESC treated for 24hr with 1µg/mL Dox to induce emGFP-PreScission-RBM15 expression, as well as to untreated cells for control-IP. To do so, 115µg of nanobodies coupled to Dynabeads were added to 18mg of mESC nuclear extract for 4 hrs at 4°C, in a total volume of 8mL IP buffer (350mM NaCl, 10% glycerol, 20mM Hepes pH7.9, 0.5mM EDTA, 0.2% Tween20, 0.5mM DTT and 1X Complete EDTA-free Protease inhibitor (Roche)). The beads were then washed six times with IP buffer containing 0.4% Tween 20, then once with PreScission buffer (50mM Hepes pH7.9, 150mM NaCl, 1mM EDTA, 1mM DTT and 1X Protease inhibitor). Beads were finally resuspended in a total of 160µL PreScission buffer supplemented with 0.6µL PreScission Protease (GE Healthcare #27-0843-01) and incubated overnight at 4°C while rotating. The elution mix was then adjusted to 0.05% SDS and incubated 10min at 10°C while shaking. The eluate was finally collected for SDS-PAGE, western-blot and mass-spectrometry analyses. Western blot analysis was carried out as detailed previously ( Nesterova *et al.*, 2019), with samples separated using a polyacrylamide gel and transferred onto PVDF membrane by semi-dry transfer. Membranes were blocked in TBS-T containing 5% w/v Marvell milk powder. Blots were incubated overnight at 4 ^°^C with either anti-Ring1B (1:3000, purified from hybridoma cells, gift from H. Koseki) or anti-RBM15 (1:3500, ProteinTech #10587-1-AP). After washing four times for 10 min with TBS-T, blots were incubated for 1hr with secondary antibody conjugated to horseradish peroxidase. After washing three times for 5 min with TBS-T, bands were visualised using ECL (GE Healthcare).

### Mass-spectrometry

Digestion of proteins was achieved using a Filter Aided Sample Preparation (FASP) protocol ( Wiśniewski *et al.*, 2009). Briefly, Vivacon 500 filters (Sartorius, VN01H02 10 kDa/VNCT01) were washed with 0.1% trifluoroacetic acid in 50% acetonitrile. The beads were loaded on the filter in 8 M urea in 100 mM AB for 30 minutes at rt. On-bead proteins were reduced (10 mM TCEP, 30 minutes, rt), alkylated (50 mM chloroacetamide, 30 min, rt in the dark) and washed (2 × 1 M urea in 50 mM AB). The proteins were subjected to tryptic digestion (0.2 µg enzyme, Promega, 1 M urea in 50 mM AB) overnight at 37°C.

Trypsinised peptides collected from the filtrate were dried and resuspended in 5% formic acid and 5% DMSO. LC-MS/MS analysis was carried out on an Ultimate 3000 ultra-HPLC system (Thermo Fisher) coupled to a QExactive mass spectrometer (Thermo Fisher). The peptides were trapped on a C18 PepMap100 pre-column (300 µm i.d. × 5 mm, 100 Å, Thermo Fisher) using solvent A (0.1% formic acid in water) at a pressure of 500 bar, then separated on an in-house packed analytical column (75 µm i.d. packed with ReproSil-Pur 120 C18-AQ, 1.9 µm, 120 Å, Dr. Maisch GmbH) using a linear gradient (length: 60 minutes, 15% to 35% solvent B (0.1% formic acid in acetonitrile), flow rate: 200 nl/min). Data were acquired in a data-dependent mode (DDA). Full scan MS spectra were acquired in the Orbitrap (scan range 350–1500 m/z, resolution 70000, AGC target 3 × 106, maximum injection time 50 ms). The 10 most intense peaks were selected for HCD fragmentation at 30% of normalised collision energy (resolution 17500, AGC target 5 × 104, maximum injection time 120 ms) with first fixed mass at 180 m/z.

### m6A-seq

m6A-seq was based on the method by Dominissini *et al.*, with minor modifications ( Dominissini *et al.*, 2013). Briefly, total RNA was extracted from pre-plated ES cells, inducing Xist by adding Dox for 24 hours, under reducing conditions using the Trizol reagents and then subjected to DNase I treatment as per the manufacturers’ instructions. RNA was fragmented by incubation for 6 min at 94°C in thin-walled PCR tubes with fragmentation buffer (100 mM Tris-HCl, 100 mM ZnCl _2_). Fragmentation was quenched using stop buffer (200 mM EDTA, pH 8.0) and incubation on ice, before ensuring the correct size (~100 bp) using RNA Bioanalyzer. Approximately 300 ug of total RNA was incubated with 12.5 ug anti-m6A antibody (Synaptic Systems, 202 003), RNasin (Promega), 2 mM VRC, 50 mM Tris, 750 mM NaCl and 5% Igepal CA-630 in DNA / RNA low-bind tubes for 2 hrs before m6A-containing RNA was isolated using 200 ul Protein A magnetic beads per IP (pre-blocked with BSA). After 2 hour incubation, extensive washing (1x IP buffer [10mM Tris-pH7.4, 150mM NaCl, 0.1%NP-40], 2x LowSalt buffer [50mM Tris-pH7.4, 50mM NaCl, 1mM EDTA, 1%NP-40, 0.1%SDS], 2x HighSalt buffer [50mM Tris-pH7.4, 1M NaCl, 1mM EDTA, 1% NP-40, 0.1%SDS], 1xIP buffer) were carried out to remove the unspecific binding. 6.7 mM m6A (Sigma) was used to elute RNA from the beads. Input and eluate samples, together with (ThermoFisher) were EtOH co-precipitated, quantitated and pooled as libraries generated using (Illumina) according to manufacturer’s instructions, but skipping the fragmentation step. 75 bp single reads were obtained using Illumina NextSeq500 sequencer.

### Data analysis

***Chromatin RNA-seq and data analysis.*** Chromatin RNA was extracted from one confluent 15 cm dish of pre-plated, feeder free mESCs as described in detail by ( Nesterova *et al.*, 2019), quantified and 1µg of RNA used for library preparation using the Illumina TruSeq stranded total RNA kit (RS-122-2301) and KAPA Library Quantification DNA standards (Kapa biosystems, KK4903). Two biological replicas of each experiment were carried out and 2X 81 paired end sequencing was performed using Illumina NextSeq500 (FC-404-2002). Chromatin RNA-seq data mapping and scripts for analysis were detailed previously ( Nesterova *et al.*, 2019), with silencing quantified using the difference in allelic ratios between uninduced and induced samples, such that

$$Gene\;silencing\;\;\left(z\right)={\left[\frac{Xi}{Xi+Xa}\right]}_{Dox}-{\left[\frac{Xi}{Xi+Xa}\right]}_{NoDox}$$

The silencing degree was compared to our previously published dataset ( Nesterova *et al.*, 2019), in which we comprehensively assayed the silencing contribution for factors/pathways involved in Xist-mediated silencing and Xist elements they bound to.

***m6A-seq and data analysis.*** For conventional m6A-seq data, we first removed the rRNA reads computationally by mapping the single-end reads to the mouse rRNA build with Bowtie2 (v2.2.6) ( Langmead & Salzberg, 2012). The remaining unmapped reads were then aligned to mm10 genome by STAR (v2.5.2b) ( Dobin *et al.*, 2013) with “--single-end” mode. BigWig files were generated by Bedtools (v2.25.0) ( Quinlan & Hall, 2010), normalized to 10 million mapped reads (see *Software availability*; Wei, 2020), and visualized in IGV or UCSC browsers.

***Mass-spectrometry analysis.*** Peptide identification and quantification were performed by MaxQuant (version 1.5.0.35i) ( Cox & Mann, 2008). MSMS spectra were searched against the Mus musculus UniProt Reference proteome (Proteome ID UP000000589, retrieved 12/01/17) alongside a list of common contaminants. The search results were filtered to a 1% false discovery rate (FDR) for proteins, peptides and peptide-spectrum matches (PSM).

For the RBM15 interactome, all hits annotated as contaminants were rejected. Then, all identified hits were compared with those identified in control-IP experiment, where emGFP-PreScission-RBM15 expression was not induced. Proteins identified in both replicates and more than eight-fold enriched in emGFP-RBM15 expressing cells compared to control-IP were classified as RBM15 interactors and kept for subsequent STRING analysis ( https://string-db.org/). STRING was performed using the following settings: 'meaning network edges' = confidence, 'minimum required interaction score' = medium confidence (0.400), 'hide disconnected nodes in the network' selected, 'kmeans clustering' = six clusters.

## Results

### Role of the 5’ Xist m6A region in Xist-mediated silencing

**Figure 1.**
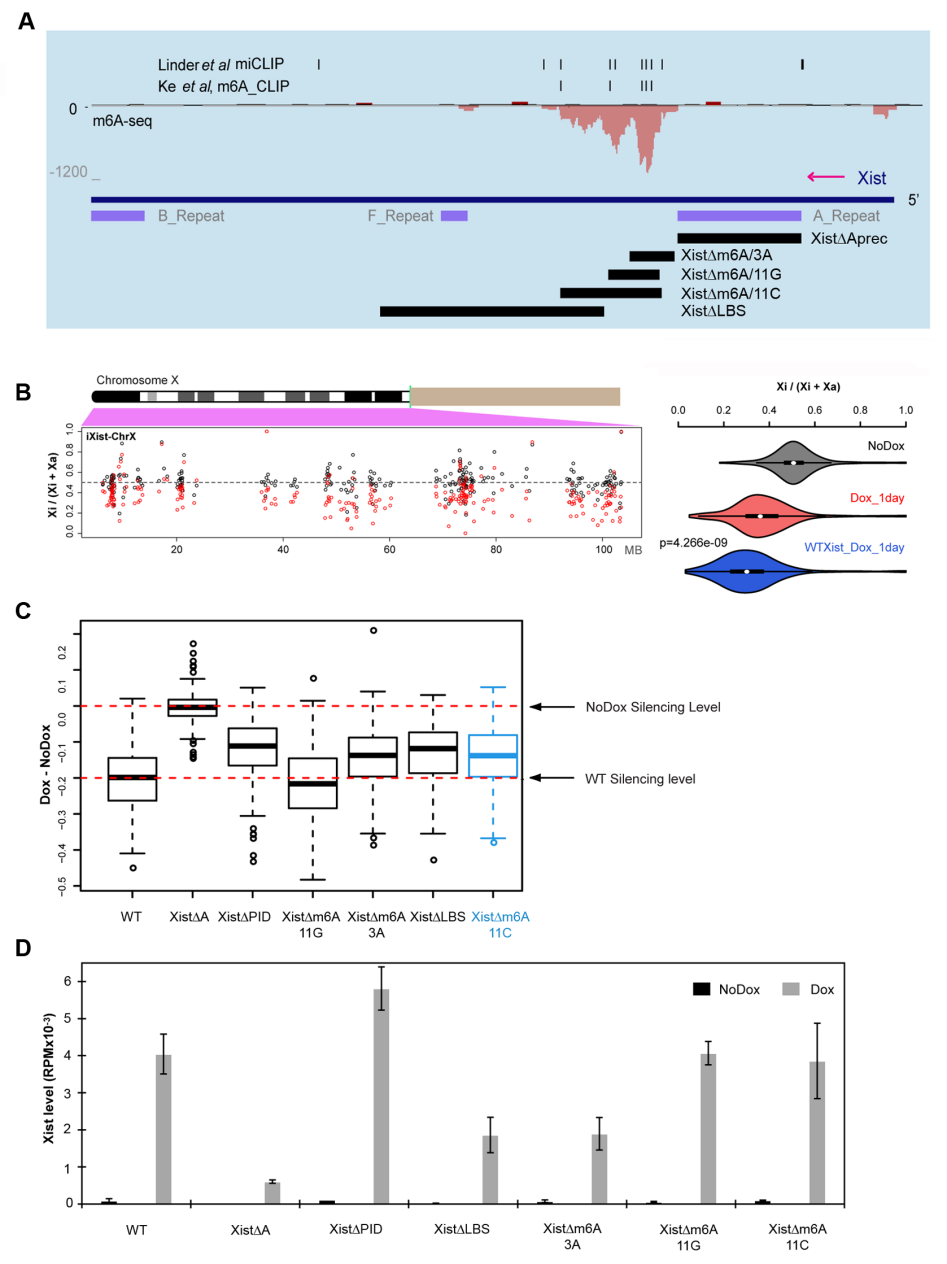
Deletion of the m6A modified region in Xist exon I has a small effect on silencing. **A**) Schematic illustrating Xist exon I surrounding the major 5’ m6A region, highlighting m6A-seq data from Nesterova *et al.*, 2019, the location of individual m6A consensus sites identified by Linder *et al.*, 2015 and Ke *et al.*, 2017 (indicated by black vertical lines above), Xist tandem repeat sequences A, F and B, and Xist deletions analysed in this study or in Nesterova *et al.*, 2019. **B**) Silencing efficiency (Xi/(Xi+Xa)) for individual informative X-linked genes in XistΔm6A/11C mESCs either with (red) or without (black) Dox induction of Xist for one day (left), with violin plots (right) showing significant difference in silencing in XistΔm6A/11C compared to wild-type (WT) mESCs (Wilcoxon test). Number of genes included in the analysis = 267. **C**) Boxplots indicating silencing efficiency of XistΔm6A/11C mESCs compared to other Xist mutants analysed in Nesterova *et al.*, 2019. Red dashed lines indicate the no Dox level of chrRNA reads from the Xi compared to WT silencing. Number of genes included in the analysis = 249. **D**) The level of Xist RNA in reads per million (RPM) in the indicated cell lines with or without one day of Dox induction.

In recent work we determined the contribution of m6A to Xist RNA silencing function by analysing mESC lines with gene knockouts for the METTL3/14 complex subunits METTL3, WTAP, and RBM15 ( Nesterova *et al.*, 2019). Additionally, because of confounding effects from m6A loss of function genome wide, we analysed overlapping deletions within the major m6A peak at the 5’ end of the Xist transcript ( Figure 1A, XistΔm6A/3A and XistΔm6A/11G), in iXist-ChrX, an interspecific (M. castaneus x 129S XX) mESC cell line with a Dox inducible Xist transgene on one X chromosome (129S allele). XistΔm6A/3A resulted in a small deficit in Xist-mediated silencing whereas XistΔm6A/11G had no effect at all.

As both previously described deletions of the 5’ m6A region remove only a subset of the m6A consensus sites, it is possible that m6A at retained sites mask a more severe effect on silencing. To investigate this possibility, we derived a new cell line with a deletion, XistΔm6A/11C, that encompasses the entire 5’ m6A region, as defined by m6A-seq analysis ( Figure 1A) ( Coker *et al.*, 2019; Nesterova *et al.*, 2019). We then assessed allelic silencing using ChrRNA-seq in XistΔm6A/11C relative to wild-type mESCs one day after inducing Xist RNA expression. As shown in Figure 1B and 1C, XistΔm6A/11C resulted in a small reduction in silencing efficiency. The magnitude of the effect was significant relative to XistΔm6A/11G, which we previously reported to have no effect, and similar to that seen for the XistΔm6A/3A and the 3’ deletion XistΔLBS, with which it partially overlaps ( Figure 1A,C). The reduced silencing efficiency was apparent for genes across the whole of the Xi ( Figure 1B). Overall levels of Xist RNA following induction, as extrapolated from ChrRNA-seq data, were similar in XistΔm6A/11C and WT cells ( Figure 1D).

### The 5’ Xist m6A region is required for transcription from the endogenous Xist promoter

**Figure 2.**
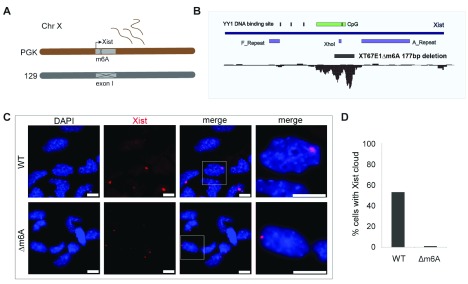
A deletion within the Xist 5’ m6A region blocks differentiation-induced Xist upregulation in an XX mESC model. **A**) Schematic indicating that XT67E1 interspecific (PGK x 129) XX mESCs have a large Xist exon I deletion on the 129 allele such that differentiation-induced Xist expression from the endogenous promoter occurs only from the PGK allele. ( **B**) In XT67E1Δm6A cells, a 177bp deletion was generated within the 5’ m6A region on the PGK allele. Schematic illustrates Xist exon I surrounding the 5’m6A region highlighting the location of the A-repeat, three previously characterised YY1 binding sites, and a XhoI restriction enzyme site (see text). An additional putative YY1 site located within the exon 1 CpG island (CpG) is deleted on XT67E1Δm6A allele. **C**) Examples of Xist RNA-FISH signal (red) in unmodified XT67E1 cells (WT), and XT67E1Δm6A (Δm6A) deletion, after four days of retinoic acid-induced ES cell differentiation. Selected areas (white box) are magnified in far-right panels. Scale bars represent 10 μm. DNA was counterstained with DAPI (blue). **D**) Xist RNA-FISH from **C**) quantified. The mean number of cells with an Xist RNA cloud was derived from two replicates with n = 100 cells scored per sample.

In the course of investigating the function of the Xist 5’ m6A region, we generated a deletion in a previously described XX mESC line, XT67E1, in which Xist expression is driven from the endogenous promoter in response to cell differentiation ( Penny *et al.*, 1996). In XT67E1 cells, a large deletion on the 129 allele, encompassing the Xist promoter and most of Xist exon I, enforces expression solely from the intact PGK Xist allele ( Figure 2A). We used CRISPR/Cas9 mediated mutagenesis to delete a 177bp region spanning the Xist 5’ m6A region from the intact Xist allele in XT67E1 XX mESCs, referred to herein as XT67E1Δm6A ( Figure 2B). Unexpectedly, XT67E1Δm6A cells failed to upregulate Xist gene expression upon differentiation, as determined by the absence of Xist RNA clouds using RNA FISH analysis ( Figure 2C and 2D).

Previous work has reported an important role for the Xist A-repeat located at the 5’ end of the transcript in Xist expression from the endogenous promoter ( Hoki *et al.*, 2009; Royce-Tolland *et al.*, 2010), and whilst the 177bp deletion we characterise lies immediately downstream of the A-repeat, it is located within the region that was deleted in the aforementioned studies. Specifically, the XhoI restriction enzyme site highlighted in Figure 2B demarcates the 3’ limit of the deletion generated in Royce-Tolland *et al.* (2010), whilst the deletion described by Hoki *et al.* (2009) extends a small distance further 3’. Our results therefore suggest that the 5’ m6A region overlaps with the major Xist enhancer located in exon I that in a previous study was reported to include a cluster of YY1 binding sites in a region 4–600 nucleotides 3’ of the A-repeat ( Figure 2B) ( Makhlouf *et al.*, 2014). We note that a consensus binding site for YY1 is located within the 177bp deletion ( Figure 2B).

### The Xist A-repeat is required for deposition of m6A over the Xist 5’ m6A region

**Figure 3.**
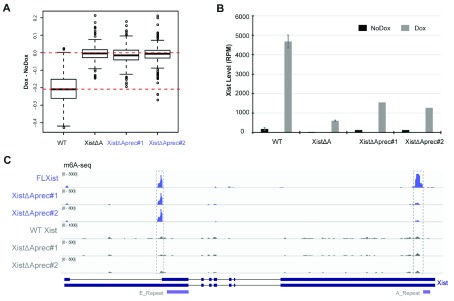
The A-repeat is required for m6A deposition at the 5’m6A region. **A**) Boxplots illustrating abrogation of Xist-mediated silencing in replicate (#1 and #2) of precise A-repeat deletion (XistΔAprec) mESC lines compared to wild-type (WT) and XistΔA mESCs described in Nesterova *et al.*, 2019. Allelic silencing (Dox-NoDox) was determined using ChrRNA-seq after one day of Dox induced Xist expression. **B**) Levels of Xist RNA in cell lines as indicated determined from chrRNA-seq data and expressed as reads per million (RPM). **C**) Genome browser snapshot showing m6A-seq data for the Xist gene in WT, ΔAprec#1 and #2 mESC lines as indicated. Input (black) and m6A IP (blue) tracks are included. Xist gene structure with the location of the A-repeat and E-repeat indicated is shown below.

Although the Xist 5’ m6A region lies downstream of the Xist A-repeat, recruitment of the m6A complex at this site has been linked to the RBP RBM15/15B, which in human XIST binds specifically within the A-repeat, as determined by iCLIP-seq ( Patil *et al.*, 2016). To directly test the requirement for the A-repeats in Xist 5’ m6A deposition in mouse, we used CRISPR/Cas9 mediated homologous recombination in iXist-ChrX XX mESCs to generate a precise deletion that removes the A-repeats but leaves all other sequences, including the m6A region, intact, referred to herein as XistΔAprec ( Figure 1A). Induction of Xist RNA in XistΔAprec mESCs revealed near complete abrogation of Xist-mediated silencing ( Figure 3A), as we reported previously using the larger XistΔA deletion ( Nesterova *et al.*, 2019). Levels of Xist RNA after induction in XistΔAprec mESCs were significantly reduced compared to wild-type iXist-ChrX mESCs ( Figure 3B), again mirroring the phenotype observed in the XistΔA mESCs ( Nesterova *et al.*, 2019). To determine the effect on m6A deposition we induced Xist RNA expression and performed m6A-seq. As shown in Figure 3C, m6A deposition in the Xist 5’m6A region was entirely lost. Equivalent results were obtained using an independently derived XistΔAprec cell line ( Figure 3A–C). Importantly, m6A deposition was unaffected at the 3’ region in Xist exon VII. Our findings confirm that the Xist A-repeat is required to recruit the m6A complex for m6A deposition at the Xist 5’ m6A region, presumably linked to RBM15/RBM15B binding.

### Proteins that interact with RBM15

**Figure 4.**
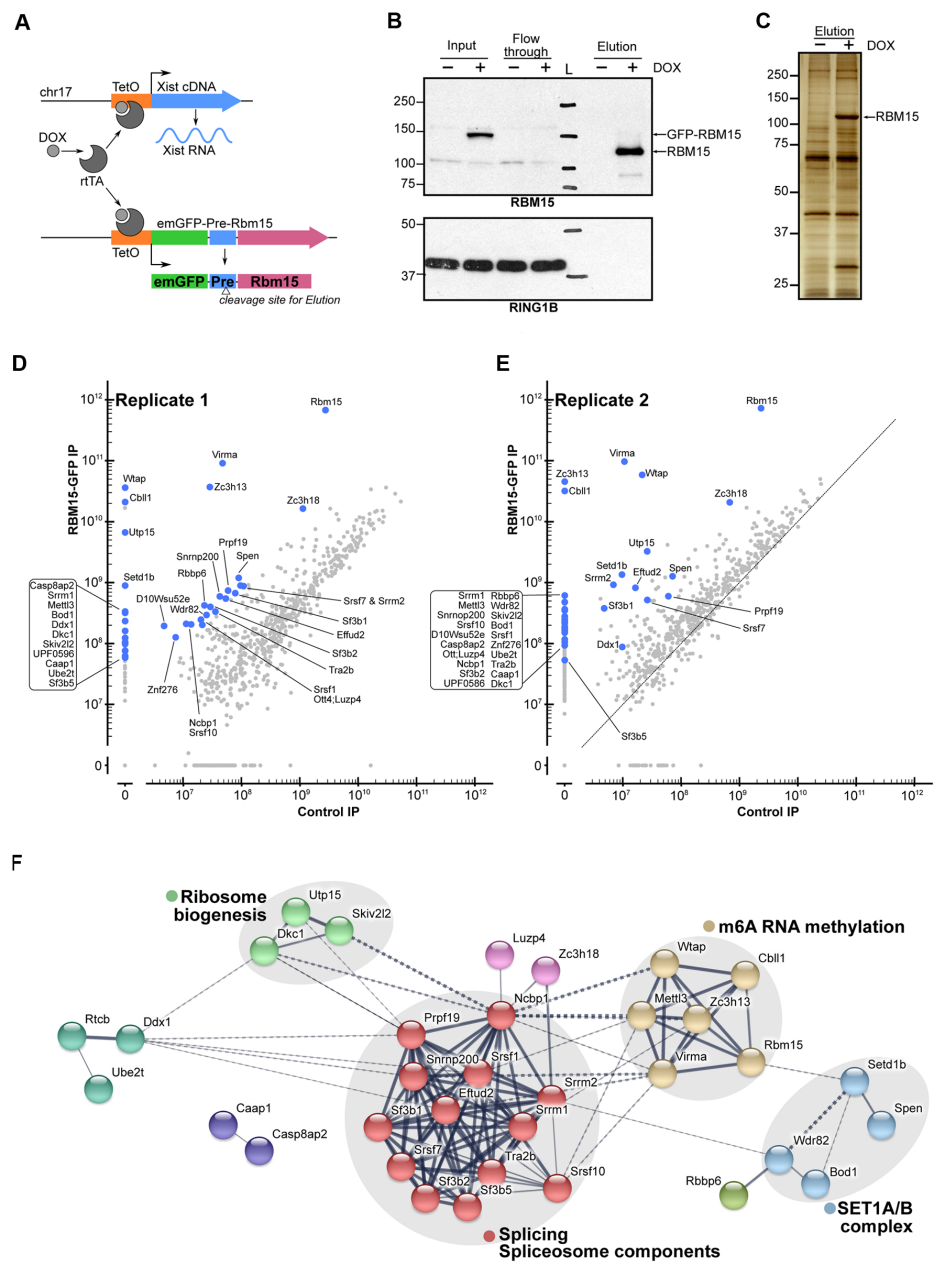
The RBM15 interactome in mESCs. **A**) Schematic illustrating strategy for expressing emGFP-tagged RBM15. Constitutively expressed Tet activator protein (rtTA) activates the TetO promoters driving both Xist cDNA and RBM15-GFP transgenes in the presence of Dox in mESCs derived from XY mESCs with inducible Xist transgene on Chr 17 described previously ( Tang *et al.*, 2010). The emGFP and RBM15 moieties are separated by a Pre proteolytic cleavage site. **B**) Western blot analysis of input and elution fractions from benzonase-treated ES cell nuclear extract, indicating the purification of RBM15 and efficient elution following cleavage of the GFP tag. RING1B was used as a loading control for input and flow-through samples. **C**) A silver stained gel of eluted proteins from mESC extracts in the absence (-) or presence (+) of Dox. Molecular weights (M _r_) are indicated in kDa. **D**) and **E**) Results of MS/MS analysis of replicate experiments highlighting those proteins that are enriched in both replicates. **F**) String analysis of RBM15 interactors highlighting that related proteins include subunits of the METTL3/14 and SET1A/B complex, and also components of the spliceosome and factors associated with ribosome biogenesis. Line thickness indicates the strength of data supporting the networks. Nodes are coloured based on known molecular functions.

The link between RBM15 and the m6A complex initially came from a proteomic analysis of interactors of the m6A regulatory subunit WTAP ( Horiuchi *et al.*, 2013) and was then confirmed in reciprocal co-immunoprecipitation experiments ( Patil *et al.*, 2016). However, RBM15 has also been shown to interact with other factors, notably the histone methyltransferase SET1B ( Lee & Skalnik, 2012), and to date there has been no unbiased analysis of the RBM15 interactome. To address this, we transfected a Dox inducible Rbm15-emGFP transgene into the previously described 3E XY mESC line, which carries a Dox inducible Xist transgene on chromosome 17 ( Tang *et al.*, 2010) ( Figure 4A). Co-induction of Xist and Rbm15 transgene expression resulted in emGFP-RBM15 fusion protein expression, allowing RBM15 and associated proteins to be purified using bead-coupled GFP-nanobodies ( Figure 4B and 4C). A proteolytic cleavage site between RBM15 and GFP was used to elute purified proteins ( Figure 4A–C), which, after benzonase treatment, were identified using MS/MS ( Figure 4D–F). Proteins purified from cells in the absence of Dox induction provided a negative control.

The RBM15 interactome analysis is summarised in Figure 4F. We found strong enrichment of proteins of the core METTL3/14 complex and associated proteins, including WTAP, CBLL1, ZC3H13, KIAA1429. We also identified enrichment of several subunits of the SETD1B complex (SETD1B, RBBP6, WDR82). In addition, we found several abundantly represented proteins of potential interest. These include proteins associated with the spliceosome (components of U2 and U5 snRNPs), and factors involved in ribosome biogenesis. Finally, we detected enrichment of the RBM15-related SPOC domain protein SPEN. Further studies are required to ascertain if these latter enrichments are attributable to direct interactions with RBM15 or indirect interactions, for example co-purifying with either SETD1B or the METTL3/14 complex.

## Discussion

Deletion of the 5’ m6A region in Xist RNA reported here has a small effect on Xist-mediated gene silencing, clarifying inconsistencies from prior work analysing partial deletions of this region and/or loss of function of METTL3/14 complex subunits. There are some remaining caveats: the 3’ m6A region located in Xist exon VII is retained, and although it is located a significant distance away from regions of the Xist transcript implicated in Xist-mediated silencing, a redundant role with the 5’ m6A region cannot be entirely ruled out. It should also be noted that deletion of the 5’ m6A region may affect Xist function independently of the m6A modification and accordingly, the small silencing deficit that we observe represents the maximum contribution of m6A within this region.

The mechanism through which m6A on Xist RNA facilitates silencing is uncertain, with possibilities including a role for the m6A binding protein YTHDC1 in recruitment of silencing factors, as suggested previously ( Patil *et al.*, 2016), a role in establishing Xist RNA architecture so as to enable silencing/localisation of Xist RNA, or a role in the Xist RNA metabolism, for example regulating Xist RNA stability/turnover, as has been suggested for cytoplasmic mRNAs ( Ke *et al.*, 2017).

Our observation that the 5’ m6A region overlaps with DNA elements essential for activation of the Xist promoter provides an explanation for prior studies which reported that the A-repeat is required for Xist gene activation, as the deletions analysed in these studies also included much of the 5’ m6A region. We note that this region includes a putative binding site for the transcription factor YY1 (and/or the closely related transcription factor REX1), consistent with the proposed importance of YY1/REX1 in Xist gene regulation ( Gontan *et al.*, 2012; Makhlouf *et al.*, 2014). Further studies are required to determine if this single site is essential for the function of the Xist enhancer.

We find that a precise excision of the Xist A-repeat region abolishes m6A deposition at the Xist 5’ m6A region, but not at the Xist 3’ m6A region. This observation is consistent with RBM15 binding to the A-repeat, promoting localised deposition of m6A through recruitment of the METTL3/14 complex. Consistent with this conclusion, iCLIP analysis identified the A-repeat of human XIST RNA as the major site for RBM15 binding in XIST ( Patil *et al.*, 2016). Our observations thus lend support to the proposal that RBM15 (and presumably RBM15B), confer sequence specific targeting of the m6A complex mRNA, including Xist.

Our proteomic analysis of RBM15 in mESCs identified several associated factors/complexes in addition to those linked to the METTL3/14 complex. Most notably we observed several subunits of the SET1B histone methyltransferase complex. This interaction was defined previously and was shown to involve the RBM15 SPOC domain. It will be interesting in the future to determine whether the SET1B interaction is mutually exclusive with binding of the METTL3/14 complex, and its importance in the context of X inactivation. Further studies are required to validate other RBM15 interaction partners identified herein and to determine if they bind RBM15 directly or indirectly.

## Conclusions

The findings reported in this study support that m6A on Xist RNA makes a small contribution to its silencing function. We confirm that the Xist A-repeat is required for m6A deposition principally in the Xist 5’ m6A region, probably via binding of RBM15. The latter is supported by identification of the METTL3/14 complex as principal RBM15 interactors. However, other factors/complexes bind to RBM15, and these may also be important in RBM15 function. Finally, we define a critical sequence element for Xist gene activation during X inactivation.

## Data availability

### Underlying data

High-throughput sequencing data (chrRNA-seq and m6A-seq) on Gene Expression Omnibus (GEO), Accession number GSE142271: https://identifiers.org/geo:GSE142271

Mass spectrometry proteomics data on PRIDE, Accession number PXD017348: https://identifiers.org/pride.project:PXD017348

Mendeley Data: The role of the Xist 5’ m6A region and RBM15 in X chromosome inactivation. http://doi.org/10.17632/56f2m8nd9h.3 ( Coker *et al.*, 2020)

This project contains the following underlying data:

- Figure 2: Raw RNA-FISH images and scoring data
- Figure 4: Raw western blot and gel data

### Extended data

Mendeley Data: The role of the Xist 5’ m6A region and RBM15 in X chromosome inactivation. http://doi.org/10.17632/56f2m8nd9h.3 ( Coker *et al.*, 2020)

This project contains the following extended data:

- Methods: Plasmid sequences

Data are available under the terms of the Creative Commons Attribution 4.0 International license (CC-BY 4.0).

## Software availability

Source code available from: https://github.com/guifengwei/XCI/blob/master/Generate_BigWig_from_RNA_seq_Bam_mm10.sh

Archived source code at time of publication: https://doi.org/10.5281/zenodo.3657009 ( Wei, 2020)

License: BSD-2-Clause
