# Supplementary material for: The role of the Xist 5’ m6A region and RBM15 in X chromosome inactivation
Source: Wellcome Open Res. 2020 Feb 17;5:31. [Version 1] doi: 10.12688/wellcomeopenres.15711.1 (PMC7097882; doi:10.12688/wellcomeopenres.15711.1)
Supplement: Peer review history

# Peer review: Reviewer response for version 1

**DOI:** 10.21956/wellcomeopenres.17220.r37945

**Published:** 2020-03-18

**Authors:** Claire Rougeulle

**Affiliations:** 1 Epigenetics and Cell Fate, CNRS, University of Paris, Paris, France

The manuscript by Coker *et al*. follows up a study by the same group (Nesterova *et al*. Nat. Comm 2019 ^1^) that revealed a much weaker contribution of the RNA methylation pathway to *Xist*-mediated silencing  than previously reported (Patil *et al.* Nature 2016 ^2^). The manuscript reports several pieces of information related to the RBM15 complex and its link to *Xist* and chromosome silencing which confirm previous findings and open new avenues.

 One important finding of Nesterova *et al.* ^1^ was the fact that ES cells permanently lacking a functional Rbm15/m6A-MTase complex could not be generated. To circumvent this limitation, the authors went on deleting regions of *Xist* in which m6A is detected. This approach has the advantage of avoiding indirect action of m6A depletion transcriptome-wide, but the disadvantage of assessing the role of a DNA/RNA region rather than of the modification of its RNA counterpart; deletion of this region might prevent binding of interactors, affect 2D structure of the RNA etc.

 By generating a deletion within the Xist 5’ region that was slightly larger than the one reported previously, the authors first find that the 5’ Xist m6A region has only a moderate effect on Xist-mediated silencing, which reinforces their previous conclusion.

 In addition, by generating this mutation not only in an artificial context in which X-inactivation is induced in undifferentiated ES cells thanks to an inducible *Xist* allele, but also in a more physiological setting (the XT67E1 cell line), the authors reveal a role for 5’ *Xist* m6A region in *Xist* up-regulation during differentiation (it would be useful to be able to compare on the same figure the position of the XT67E1Dm6A and that of other deletions). The authors conclude as to a function for this region in *Xist* transcription, although a role on *Xist* stabilization/accumulation cannot be excluded, as this is based solely on RNA-FISH analysis. Related to this comment, the difference in the number of *Xist* positive cells between WT and XT67E1Dm6A is not striking from the image shown (Figure 2C), and thus does not illustrate appropriately the counting provided as Figure 2D. The size of the *Xist* cloud seems different though and should be measured.

 The authors further confirm that the *Xist* A-repeat, that lies just upstream of the methylated region, is required for m6A deposition to the 5’, but not the 3’ region. Finally, the authors provide a list of factors interacting with RBM15, identified in a context of co-induction of RBM15 and Xist. This sets the basis for future analyses, but the rational of this experimental setting and whether it results in an enrichment of Xist-linked RBM15 interactors is not shown nor discussed.

I confirm that I have read this submission and believe that I have an appropriate level of expertise to confirm that it is of an acceptable scientific standard.

## References

[1] : Systematic allelic analysis defines the interplay of key pathways in X chromosome inactivation. *Nature Communications*.2019;10(1) : 10.1038/s41467-019-11171-3 10.1038/s41467-019-11171-3 PMC663539431311937

[2] : m6A RNA methylation promotes XIST-mediated transcriptional repression. *Nature*.2016;537(7620) : 10.1038/nature19342 369-373 10.1038/nature19342 PMC550921827602518

# Peer review: Reviewer response for version 1

**DOI:** 10.21956/wellcomeopenres.17220.r37943

**Published:** 2020-03-16

**Authors:** Carolyn Brown

**Affiliations:** 1 Department of Medical Genetics, Molecular Epigenetics Group, Life Sciences Institute, The University of British Columbia, Vancouver, BC, Canada

This article is a follow-up to the 2019 Nesterova *et al.*, Nature Communications paper “Systematic allelic analysis define the interplay of key pathways in X chromosome inactivation”; which had reported that the contribution of RBM15/15b to Xist-induced silencing was minor relative to the impact of SPEN, and also the polycomb pathways. This is in contrast to reports that argued RBM15/15b to be critical for X inactivation, and could be rescued by tethering of the m6A reader YTHDC1 (Patil *et al.*, 2016 Nature ^1^). This work assesses the contribution of m6A to X inactivation by deletion of regions around the A repeat, and then further explores the interactome of RBM15.

 These new deletions are compared with constructs from the prior paper, and I felt that this could be clarified by colour-coding the new deletions – as was done in panel 1C. Additionally, as panel 1C aggregates results from 249 genes, it seems that a p-value could be generated (as done for panel 1B) to statistically test the difference from both wild-type and the delta-A construct.

 Surprisingly, despite the expression of these constructs being driven by an inducible promoter, the expression level of Xist was seen to be reduced. This region is described as overlapping a previously described enhancer.

 While the constructs described above used an inducible Xist construct, the paper also examines the impact of a similar deletion on the endogenous gene in a mouse ES cell line that carries an Xist deletion to force Xist expression and silencing from the “PGK” chromosome (Figure 2). Continuity of the labelling from Figure 1 (including the Xist transcription direction on Figure 2 and the CpG island and deletion sizes on Figure 1) would be helpful. Additionally, perhaps inclusion of CTCF sites would be informative given the previous reports of CTCF being involved in the regulation of Xist?

 The assessment of these cells relies upon RNA-FISH for examining Xist levels, and I believe would be complemented well by q-RT-PCR, as failure to localize Xist may not reflect decreased expression levels.

 Switching back to the inducible construct model, a precise deletion of only the A repeat region was shown to have similar impact on Xist expression level, but importantly to only impact m6A deposition at the 5’ end of Xist, with the m6A distal to the E repeat remaining high. 

 The manuscript then shifts to the proteomic analysis of the RBM15 interactome. Interestingly, this approach, which uses a Dox-inducible Rbm15-emGFP transgene is also performed in the presence of the Dox-inducible Xist construct. It would have been interesting to know if any of the observed partners were Xist-dependent (such as SPEN) and might not be seen to interact in the absence of Xist. 

 The study concludes that m6A has only a small contribution to silencing function; however, to be accurate the conclusion would be that m6A at the 5’ end of Xist has only a small contribution. The authors note the caveat that 3’ m6A remains; and I believe that this limits their ability to conclude that it is only playing a minor role.

I confirm that I have read this submission and believe that I have an appropriate level of expertise to confirm that it is of an acceptable scientific standard, however I have significant reservations, as outlined above.

## References

[1] : m6A RNA methylation promotes XIST-mediated transcriptional repression. *Nature*.2016;537(7620) : 10.1038/nature19342 369-373 10.1038/nature19342 PMC550921827602518

# Peer review: Reviewer response for version 1

**DOI:** 10.21956/wellcomeopenres.17220.r37944

**Published:** 2020-03-09

**Authors:** Takashi Sado, Saya Ichihara

**Affiliations:** 1 Department of Advanced Bioscience, Graduate School of Agriculture, Kindai University, Nara, Japan

To extend their previous work by Nesterova *et al.* (2019) ^1^, the authors deleted an entire sequence of the 5’ Xist m6A region in two different female ESC lines and studied the effect on Xist-mediated chromosome silencing. At variance with previous work by Patil *et al.* (2016) ^2^, the mutated Xist RNA induced from the dox-regulatable allele had only a minor effect causing a small reduction in silencing efficiency. On the other hand, a simple deletion of the m6A region in the Xist gene resulted in a failure in the upregulation of the mutated allele upon differentiation, suggesting a role of the deleted region in the activation of the endogenous promoter as a cis element. They also showed that Xist RNA lacking a precise sequence of the A-repeat had lost m6A modification over the 5’ m6A region. This is consistent with a proposed role of the A-repeat as a module for RBM15/RBM15B binding and subsequent recruitment of METTL3/14 complex on Xist RNA. In addition, they found that levels of Xist RNA induced from the mutated allele lacking the A-repeat was significantly lower than those of wild-type Xist RNA. Finally, they showed that RMB15 interacted with not only m6A complex such as METTL3/14 complex but also factors involved in histone modification and RNA metabolism. The findings provided here support the idea that RBM15 biding to the A-repeat recruits the m6A complex to the 5’ Xist m6A region and suggest a role of m6A modification in silencing function of Xist RNA and its metabolism.

 Although the findings of the study may be limited, the experiments were well conducted and reliable, and therefore, the results provided here are worth sharing among the community of X inactivation and related fields. I have some comments and questions, however, which I suggest the authors to address.

 Deletion of the precise A-repeat region resulted in a reduction in levels of Xist RNA after induction. It is not clear if this comes from an inefficient induction by dox or a decrease in the stability of the mutated Xist RNA lacking m6A modification. I think it is important to know if m6A in the 5’ Xist m6A region is involved in the stability of Xist RNA. The authors should examine this.

 An increase in Tsix expression, if any, could also result in a reduction in levels of Xist expression by causing inefficient induction of the mutant Xist allele by dox inΔAprec mutants. I am wondering if deletion of the A-repeat affect expression of Tsix as described by Hoki *et al.* (2009) ^3^ and Tsix thus expressed has a negative impact even on the exogenous dox-inducible promoter.

 The authors seem to misunderstand the structure of the mutant alleles lacking the A-repeat previously reported. The deletion of the A-repeat generated by Royce-Tolland *et al.* (2010) ^4^ using a PCR-based targeting vector precisely lacks a sequence of the A-repeat, and it is the deletion generated by Hoki *et al. *(2009) ^3^ that the XhoI site highlighted in Figure 2B demarcates the 3’ limit of the A-repeat deletion.

We confirm that we have read this submission and believe that we have an appropriate level of expertise to confirm that it is of an acceptable scientific standard, however we have significant reservations, as outlined above.

## References

[1] : Systematic allelic analysis defines the interplay of key pathways in X chromosome inactivation. *Nature Communications*.2019;10(1) : 10.1038/s41467-019-11171-3 10.1038/s41467-019-11171-3 PMC663539431311937

[2] : m6A RNA methylation promotes XIST-mediated transcriptional repression. *Nature*.2016;537(7620) : 10.1038/nature19342 369-373 10.1038/nature19342 PMC550921827602518

[3] : A proximal conserved repeat in the Xist gene is essential as a genomic element for X-inactivation in mouse. *Development*.2009;136(1) : 10.1242/dev.026427 139-46 10.1242/dev.026427 19036803

[4] : The A-repeat links ASF/SF2-dependent Xist RNA processing with random choice during X inactivation. *Nat Struct Mol Biol*.2010;17(8) : 10.1038/nsmb.1877 948-54 10.1038/nsmb.1877 20657585PMC4336797
